# Evaluation of an E2-based indirect ELISA for the serological differentiation of ovine Italy pestivirus from classical swine fever virus in pigs

**DOI:** 10.3389/fvets.2026.1847235

**Published:** 2026-06-16

**Authors:** Manuel Corsa, Giulia Pezzoni, Anna Castelli, Roberto Benevenia, Matteo Ricchi, Monica Giammarioli, Llilianne Ganges, Denise Meyer, Paul Becher, Enrica Sozzi

**Affiliations:** 1 Istituto Zooprofilattico Sperimentale della Lombardia e dell’Emilia-Romagna “Bruno Ubertini” (IZSLER), Brescia, Italy; 2 Istituto Zooprofilattico Sperimentale dell’Umbria e delle Marche “Togo Rosati” (IZSUM), Perugia, Italy; 3 WOAH Reference Laboratory for Classical Swine Fever, IRTA-CReSA, Barcelona, Spain; 4 IRTA, Programa de Sanitat Animal, Centre de Recerca en Sanitat Animal (CReSA), Bellaterra, Barcelona, Spain; 5 Institute of Virology, University of Veterinary Medicine Hannover, Foundation, Hannover, Germany

**Keywords:** classical swine fever virus, E2 glycoprotein, indirect ELISA, ovine Italy pestivirus, pigs, recombinant protein, serological differentiation

## Abstract

The genus *Pestivirus* includes several species responsible for economically important diseases in domestic and wild animals. In 2017, a novel ovine pestivirus (ovIT PeV) was isolated from aborted lamb fetuses and molecular analysis showed a genetic distinction from known *Pestivirus* species with the closest phylogenetic relationship to classical swine fever virus (CSFV, *Pestivirus suis*). This close similarity is concerning because CSF is a notifiable disease to the World Organization for Animal Health (WOAH) and is subject to eradication campaigns. Moreover, ovIT PeV can infect pigs and induce an early strong immune response that can cause false-positive results in CSF antibody ELISAs used for CSF surveillance. Our study aimed to develop an indirect ELISA (iELISA) to serologically distinguish ovIT PeV from CSFV in pigs. Hence, we expressed the recombinant E2 glycoproteins of ovIT PeV and CSFV (strain Diepholz) in mammalian cells. Two E2 iELISAs were evaluated using a panel of CSFV, ovIT PeV and negative pig sera. Each serum showed higher antibody titres in the iELISA with the homologous antigen rather than the heterologous one. This study highlighted the effectiveness of using virus-specific antigens and comparing antibody titres when assessing infections with serologically cross-reactive viruses.

## Introduction

The genus *Pestivirus* within the family *Flaviviridae* (25) encompasses a wide diversity of a growing number of established virus species and unclassified, so-called atypical pestiviruses, which can have significant economic impact on livestock species, especially ruminants and swine. Some of the historic pestiviruses have been known for decades, such as Bovine viral diarrhoea virus (BVDV) type 1 (*Pestivirus bovis*) and type 2 (*Pestivirus tauri*), classical swine fever virus (CSFV, *Pestivirus suis*) and Border disease virus (BDV, *Pestivirus ovis*) (1), while several other pestiviruses have been identified in domestic animals and a growing number of wild animal hosts during the last two decades (1, 2). Pestiviruses are endemic in most regions of the world and spread by direct and indirect contact from infected animals to susceptible animals resulting in subclinical infections or a variety of clinical signs such as hemorrhagic syndromes, abortion or embryonic death, diarrhoea, respiratory and wasting diseases (3). Pestiviruses are enveloped viruses with a linear positive-sense single stranded (ss) RNA genome of about 12 kb, comprising a single open reading frame translated into one large polyprotein, which is further processed by host and viral proteases. Pestiviruses encode eight non-structural proteins (NSPs), involved in viral maturation and polyprotein processing, and four structural proteins (SPs). Among NSPs, NS3 is highly immunogenic and highly conserved among pestiviruses (4), thus antibodies targeting NS3 exhibit a high degree of cross-reactivity (5). The SPs include an internal nucleocapsid core protein (C) and three envelope glycoproteins (E^rns^, E1 and E2). The envelope glycoproteins E^rns^ and E2 are the most immunodominant and virus-specific proteins of pestiviruses (6), in particular E2 is able to elicit the major neutralizing antibody response (7). Nevertheless, even in the case of the envelope glycoproteins serological cross-reactions can still occur (8–10, 23).

In 2019, the novel ovine pestivirus (ovIT PeV) was detected in aborted lambs in northern Italy (11). Experimental infections of pregnant ewes and domestic pigs with an ovIT PeV isolate from Italy (IT/ov/1756/2017) demonstrated its viral replication and pathogenesis, the induction of immune response, the antibody protection against other pestiviruses and the capacity of this virus to infect swine (10, 12).

Classical swine fever (CSF) is a highly contagious, viral disease of swine caused by CSFV with high morbidity and mortality rates (9, 13). The most frequently used competitive CSF antibody ELISAs cannot distinguish antibodies against ovIT PeV from antibodies against CSFV and porcine serum samples collected after ovIT PeV infection were detected as false-positive due to cross-reactivity (14). In the present study, two indirect ELISAs (iELISAs) were developed using recombinant glycoprotein E2 of ovIT PeV (E2-ovIT PeV) and E2 of CSFV strain Diepholz (E2-CSFV). Antibody titers against the heterologous and homologous E2 antigens were determined using serum samples collected from pigs experimentally infected with CSFV or ovIT PeV. On the basis of the detected antibody titers, the differentiation between both pestivirus infections was evaluated. An overview of the overall experimental workflow is provided in Supplementary Figure 1.

## Materials and methods

### Cloning of E2-ovIT PeV and E2-CSFV sequences

The nucleotide sequences of the ovIT PeV and CSFV (strain Diepholz) were obtained from Genbank (Accession number: PQ197220.1 and JQ411564.1).

The nucleotide sequences of the E2-ovIT PeV (2,425–3,544 nt) and E2-CSFV (2,246–3,271 nt), excluding intra-virion and transmembrane domains, were optimized according to the human codon usage and cloned in two distinct pcDNA3.4/TOPO plasmids by GenArt tool (Thermo Fisher Scientific, Waltham, Massachusetts, USA). For proper initiation of translation in mammalian cells, a Kozak consensus sequence was inserted at the N-terminus of each construct (15). In addition, a murine Igk-chain leader sequence (16), for endogenous secretion, was added. Antigen purification was performed by a polyhistidine tag, which was fused at the C-terminus.

### Cells and proteins expression

The Expi293F cells (Thermo Fisher Scientific, Waltham, Massachusetts, USA) were grown in suspension using Expi293 Expression Medium (Thermo Fisher Scientific, Waltham, Massachusetts, USA). The cells were growth in sterile, non-baffled and vent-cap flasks in an orbital shaker at 125 rpm (orbital diameter of 19 mm) in a 37 °C incubator with ≥80% relative humidity and 8% CO_2_. The cell number and viability were evaluated using a Burker chamber and the Trypan blue for cell staining. Two 125 mL flasks with 25 mL of cell culture each were used: one for E2-ovIT PeV and another for E2-CSFV optimized plasmid. On the day of transfection the cell cultures were diluted to a final density of 3 × 10^6^ viable cells/mL. The viability of the cells of at least 95% was monitored in the experiment. The codon optimized plasmids were transiently transfected into human Expi293F cells using Expi293 Expression System (Thermo Fisher Scientific, Waltham, Massachusetts, USA) following the manufacturer’s instructions. Briefly, ExpiFectamine 293 Reagent (Thermo Fisher Scientific, Waltham, Massachusetts, USA) and one μg/mL of plasmid DNA were separately diluted in OptiMEM I Reduced Serum Medium (Thermo Fisher Scientific, Waltham, Massachusetts, USA). Following a 5 min incubation at room temperature (RT), ExpiFectamine 293 Reagent and DNA mixtures were combined and incubated for an additional 20 min at RT. The ExpiFectamine 293 Reagent-DNA-OptiMEM mixture was then added to cells, drop-wise by swirling the flask gently. Enhancer 1 and Enhancer 2 reagents (Thermo Fisher Scientific, Waltham, Massachusetts, USA) were added to transfected cultures 16–18 h post-transfection. At 3 days post-transfection, cultures were centrifuged 10 min at 90 × *g* at RT, supernatants were collected and stored at −20 °C, pellets were gently resuspended in 30 mL of fresh Expi293 Expression Medium and the cells resumed growth in the shaking incubator for another 3 days (17). Finally, the cultures were centrifuged 15 min at 300 x g at 4 °C and only supernatants were collected and stored at −20 °C.

### Proteins purification

The culture supernatants of E2-ovIT PeV and E2-CSFV collected at three and 6 days post-transfection were submitted to Immobilized Metal Affinity Chromatography (IMAC) purification. A total of 1.5 mL of Ni-NTA Agarose resin (QIAGEN, Hilden, Germany) was added to each supernatant and then left on a benchtop shaker overnight at 4 °C. After incubation, polypropylene columns (Econo-Column® Chromatography Columns, Bio-Rad, Hercules, California, USA) were loaded with each supernatant-resin mixture, the resin was packed via gravity flow to the bottom of the column forming the bed volume (750 μL). Subsequently, the so-called flow thought (FT) was collected and then the Washing Buffer (500 mM NaCl, 50 mM TrisHCl and 10% glycerol at pH 7.5 + Imidazole 20 mM), in a volume of eight times the resin bed, was loaded in the column and allowed to elute, collecting Wash 1 (W1); the procedure was repeated a second time collecting Wash 2 (W2). Finally, the Elution Buffer (500 mM NaCl, 50 mM TrisHCl and 10% glycerol at pH 7.5 + Imidazole 250 mM), in an equal volume of resin bed, was added for five times and the five elutions were collected separately (E1 to E5). The concentration of the proteins was determined by measuring the UV absorbance at 280 nm on the UV–VIS Agilent 8,453 (Agilent Technologies, Santa Clara, California, USA) spectrophotometer.

### Panel of sera and pre-screening tests

A panel of 40 porcine sera (CSF-1 to CSF-40) from 40 pigs experimentally infected with various CSFV genotypes was included in the study (Supplementary Table 1) (24). Of these, 23 samples (CSF-1 to CSF-23) originated from experimental infections conducted at the Istituto Zooprofilattico Sperimentale della Lombardia e dell’Emilia Romagna, while the remaining 17 samples (CSF-24 to CSF-40) were obtained from the collection of the EU and WOAH Reference Laboratory for CSF.

In addition 11 porcine sera from ovIT PeV experimental infection carried out by IRTA-CReSA Centre (Barcelona, Spain) (12) were used. Ten sera were collected from five pigs (ovIT PeV 1–5) at two time points each, 28 and 35 days post-infection (dpi), hereafter defined as t1 and t2, respectively. One additional serum was collected from a sixth pig at 28 dpi only (ovIT PeV 6-t1).

Furthermore, 339 sera collected from different pig farms in Lombardy in the framework of the national surveillance plan for CSF, were also tested. All samples were collected by official veterinarians as part of the Italian national plan for the surveillance of classical swine fever (CSF) in swine herds, in compliance with the national surveillance protocols.

To confirm the serological status of the animals, all the sera were tested with two ELISAs: a homemade pan-pestivirus competitive ELISA based on the recombinant NS protein 3 (NS3) and a commercial CSFV Ab test (IDEXX Laboratories, Liebfeld, Switzerland); the latter test was carried out according to the manufacturer’s recommendations while the homemade pan-pestivirus competitive NS3 ELISA is briefly described below. The anti-NS3 monoclonal antibody (Mab) 3H4 was adsorbed onto a Nunc Maxisorp microplate (Thermo Fisher Scientific, Waltham, Massachusetts, USA) diluted at 1:400 in carbonate bicarbonate buffer (15 mM Na₂CO₃ and 35 mM NaHCO₃, ph 9.6) and incubated overnight at 4 °C. The next day, three washing steps were performed with wash buffer containing PBS-0.05% Tween. The antigen-serum mixture was prepared on a pre-incubation microplate MICROLON® 200, medium binding, flat bottom (Greiner Bio-One, Monroe, NC): 50 μL of 1:2 diluted serum with 50 μL of pre-titrated NS3 recombinant antigen, diluted 1:60 in dilution buffer (1% yeast extract, PBS-0.05% Tween). After an incubation of 1 h at 37 °C, 50 μL each antigen-serum mixture was transferred to the plate with absorbed the Mab 3H4 and incubated for an additional 1 h at 37 °C. After the incubation and three washes, 50 μL of peroxidase-conjugated anti-NS3 Mab 3A3 (1:400 diluition) was added. After a final incubation for 1 h at 37 °C and three washes with wash buffer, the chromogenic substrate was added and the optical densities (OD) were read using a spectrophotometer at 492 nm. In the reaction the following controls were included: a 100% control with absence of inhibition (no serum), a negative control serum, a strong and a weak positive control serum. For each examined serum the inhibition percentage (IP) was calculated as follow (Equation 1):

$$\mathrm{IP}=100-(\frac{\text{serum}\;\mathrm{OD}\;\text{value}\;}{100\%\text{control}\;\mathrm{OD}\;\text{value}})\times 100$$ (1)

### SDS-PAGE and Western Blotting

The protein expression was checked by SDS-PAGE using NuPAGE™ Bis-Tris Mini Protein Gels, 4–12% (Invitrogen, Waltham, Massachusetts, USA) and Quick Coomassie Stain (Protein Ark, Rotherham, England), according to the manufacturer instructions.

**Figure 1:**
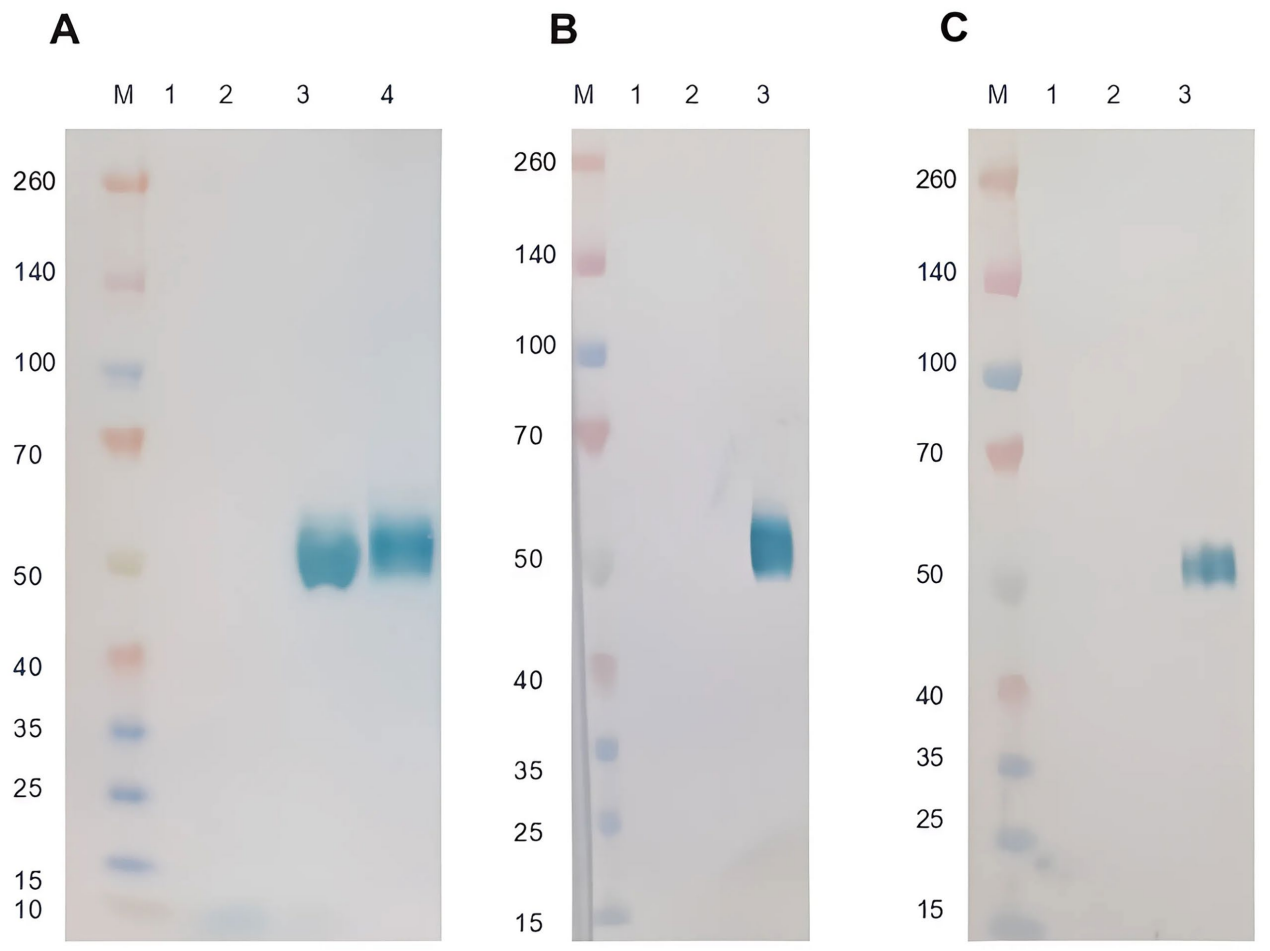
Western Blotting. **(A)** Reactivity of a penta·His Antibody against the purified recombinant proteins. Lane 1: no sample loaded; lane 2: negative control; lane 3: E2-ovIT PeV; lane 4: E2-CSFV. **(B)** Reactivity of ovIT PeV negative pool sera (lane 2) and positive pool sera (lane 3) against the E2-ovIT PeV; in lane 1 no sample was loaded. **(C)** Reactivity of CSFV negative pool sera (lane 2) and positive pool sera (lane 3) against the E2-CSFV; in lane 1 no sample was loaded. The purified recombinant proteins were loaded for a total amount of 150 ng/well. M: molecular weight marker (kDa).

Western Blotting (WB) was performed to confirm the identity of the his-tagged purified proteins. In brief, 150 ng/well of each purified protein, denatured in sodium dodecyl sulphate (SDS) and β-mercaptoethanol, was loaded and separated on precast polyacrylamide gels at 4–12% gradient concentration (NuPAGE™ Bis-Tris Mini Protein Gels). After the run, the proteins were transferred to polyvinylidene difluoride (PVDF) membranes using Trans-Blot® Turbo™ Transfer System (Bio-Rad, Hercules, California, USA), followed by blocking with 5% skim milk in PBS-0.2% Tween overnight at 4 °C. The membrane was washed three times for 10 min with PBS-0.2% Tween, then Penta·His Antibody, BSA-free (QIAGEN, Hilden, Germany) was diluted in 2% skim milk in PBS-0.2% Tween at the concentration of 0.2 μg/mL, according to the manufacturer instructions, and incubated at RT for 1 h. Following an additional three washes, the membranes were incubated with HRP-conjugated goat anti-mouse IgG Mab (1:250 dilution) for 1 h at RT. The membrane was washed and detected with Novex™ HRP Chromogenic Substrate (TMB) (Invitrogen, Waltham, Massachusetts, USA) for 5 min to let the bands develop (Figure 1A).

An additional WB of the purified proteins was performed with specific pathogen positive and negative sera used as primary antibodies to confirm the proteins identity (Figures 1B,C). The WB procedure was the same as explained above except for the primary antibody. A positive ovIT PeV porcine sera pool or a positive CSFV porcine sera pool was applied for the detection of recombinant E2-ovIT PeV and recombinant E2-CSFV. A negative porcine sera pool served as a negative control. As a secondary antibody, a homemade HRP-conjugated anti-swine IgG Mab 4B6, at 1:100 dilution, was used. All sera were diluted 1:20 in 2% skim milk and in PBS-0.2% Tween.

### Indirect enzyme-linked immunosorbent assay (iELISA) development and serum titrations

In the two developed iELISA assays, each of the recombinant proteins was adsorbed onto the microplates. The optimal protein concentration was determined through cross titration with the horseradish peroxidase-conjugated anti-swine IgG Mab 4B6 using three sera diluted at 1/50 (one serum against CSFV strain Diepholz, one serum against ovIT PeV and one serum containing no antibodies against pestiviruses). The E2-ovIT PeV and E2-CSFV were coated on microplates Nunc Maxisorp (Thermo Fisher Scientific, Waltham, Massachusetts, USA) using a two-fold serial dilution series, starting from 0.6 μg antigen/mL down to 0.002 μg antigen/mL. Antigens were diluted in carbonate bicarbonate buffer and incubated overnight at 4 °C on the plates. Wells with no antigen were used for blank values to evaluate the background signal of the sera and the conjugated Mab. After three washes with PBS -0.05% Tween buffer, the three sera were added using a 1:50 dilution in dilution buffer. After an incubation of 1 h at 37 °C, three additional washes were performed and 4B6-HRP-conjugated Mab was added using a two fold dilution series ranging from 1:100 to 1:400 in dilution buffer. Following final incubation for 1 h at 37 °C and three washes with wash buffer, 0.5 mg/mL of OPD (o-phenylenediamine) diluted in phosphate–citrate buffer (C₆H₈O₇ 0.1 M and Na₂HPO₄ 0.2 M, pH 5.6) and supplemented with 0.02% H_2_O_2_ was distributed (50 μL per well). After 10 min the reaction was stopped with 50 μL per well of 2 N sodium hydroxide. The OD was measured by spectrophotometer Multiskan ascent (Thermo Fisher Scientific, Waltham, Massachusetts, USA) at 492 nm. Afterwards, the blank OD value was subtracted from the corresponding sample OD value obtaining the “net OD.”

Once the optimal concentration of the recombinant proteins and the conjugated Mab were determined, the iELISA for end-point titration was carried out as follows: both purified proteins were absorbed on the Nunc Maxisorp plate (Thermo Fisher Scientific, Waltham, Massachusetts, USA) in alternating columns using carbonate bicarbonate buffer and incubated overnight at 4 °C. An additional column was included containing only carbonate bicarbonate buffer to define the blank value (no antigen). Three washes were performed the next day with the wash buffer (PBS-0.05% Tween). Subsequently, the sera were added in seven consecutive threefold serial dilutions starting from 1:50 (final dilution 1:109,350). For each serum, 75 μL of the initial dilution were dispensed into the first wells of three consecutive columns, and serial dilutions were generated by stepwise transfer of 25 μL from well to well down each column into 50 μL of dilution buffer already dispensed. In addition, a CSFV-positive control serum, and a negative control serum, at a fixed dilution of 1:50, were included. After an incubation for 1 h at 37 °C, the plate was washed as described above and 50 μL of the 4B6-HRP-conjugated anti-swine IgG was added using a 1:200 dilution in dilution buffer. After a final incubation for 1 h at 37 °C, three washes with wash buffer and substrate incubation, OD values were measured in the spectrophotometer and the net ODs were calculated.

For each tested serum the endpoint titre was calculated against both the recombinant antigens (18). The antibody titre was defined as the highest serum dilution at which the net OD corresponded to the established cut-off value. Endpoint titres were estimated by interpolation using a trend function based on the two OD values immediately above and below the cut-off, thereby determining the serum dilution corresponding to the cut-off OD. Titer calculation was performed twice for each serum in independent experiments to ensure reproducibility (data not shown).

To establish a discriminatory threshold between CSFV and ovIT PeV infections, the ratio of endpoint titers obtained from the two iELISAs was calculated for each serum sample as follow:

$$\frac{E2-\text{CSFV titer}}{E2-\text{ovIT}\;\mathrm{PeV}\;\text{titer}}$$ (2)

For samples with E2-ovIT PeV undetectable titer, ratio was not calculated.

### Virus neutralization assay (VNT)

Sera collected from animals infected with various CSFV genotypes (CSF-30 to CSF-40), as well as sera from ovIT PeV infected animals (ovIT PeV-1 to ovIT PeV-6), were tested in virus neutralization test (VNT) (Supplementary Table 1).

The following pestivirus were used: BVDV-1 (strain NADL), BVDV-2 (strain CS8644), CSFV (strain Diepholz) and ovIT PeV (isolate IT/ov/1756/2017).

The VNTs were performed according to the protocol described in the Manual of Diagnostic Tests for Detection of CSF, which is available on the website of the EU and WOAH reference laboratory for CSF (19).

Briefly, before testing in the VNT the serum samples were heat-inactivated at 56 °C for 30 min. Each serum sample was titrated in a twofold serial dilution starting with a 1:5 up to a 1:10,240 dilution and was subsequently incubated for 1 h with the virus suspension. Each virus was applied with a constant amount of 100 ± 0.5 log_10_ tissue infectious dose 50 (TCID_50_). Subsequently susceptible cells were added, and the assay was incubated for 72 h at 37 °C and 5% CO_2_. Porcine kidney cells (PK-15) were used for infection with the CSFV strain Diepholz, Madin-Darby bovine kidney cells (MDBK) were used for infection with the BVDV-1 strain NADL and the BVDV-2 strain CS8644, and sheep fetal thymus cells (SFT-R) for the infection with ovIT PeV (isolate IT/ov/1756/2017). Neutralizing antibody titers were determined by end-point titration. Using the Spearman-Kärber method (20, 21) and were expressed as initial sample dilution.

All VNTs were conducted in parallel at the WOAH and EU Reference Laboratory for CSF under biosafety level three conditions.

### Statistical analysis

The titres of pigs infected with ovIT PeV and CSFV, evaluated using both E2-ovIT PeV and E2-CSFV, were compared using Wilcoxon Signed Rank Test.

Inter-assay variability was assessed using positive control OD values across independent runs, while reproducibility was evaluated by comparing endpoint titres of serum samples obtained in two runs.

### Ethics approval for animal experiments

All animal procedures were conducted in accordance with relevant guidelines and regulations, and are reported in compliance with the ARRIVE guidelines.^1^ Experimental protocols involving animals were approved by the appropriate institutional and/or national ethical committees, as follows:

- Sera from animals experimentally infected with ovine Italy pestivirus (ovIT PeV) were provided by the IRTA-CReSA Centre (Barcelona, Spain). These samples were obtained following approval by the Ethical Committee of the Generalitat de Catalonia, Spain (Project Number: 10631), and in accordance with applicable European regulations.
- Serum samples from the EU and WOAH Reference Laboratory for Classical Swine Fever (CSF) were derived from experiments reported to the Specialized Department of the Animal Welfare Service of the Lower Saxony State Office for Consumer Protection and Food Safety (LAVES; Permit Number: LAVES AZ 08A 538), in accordance with the German Animal Welfare Act.
- Additional experimental sera from the Istituto Zooprofilattico Sperimentale della Lombardia e dell’Emilia Romagna originated from infections conducted in the early 1990s, in compliance with the national regulations in force at that time.

## Results

### Recombinant proteins expression, purification and characterization

**Figure 2:**
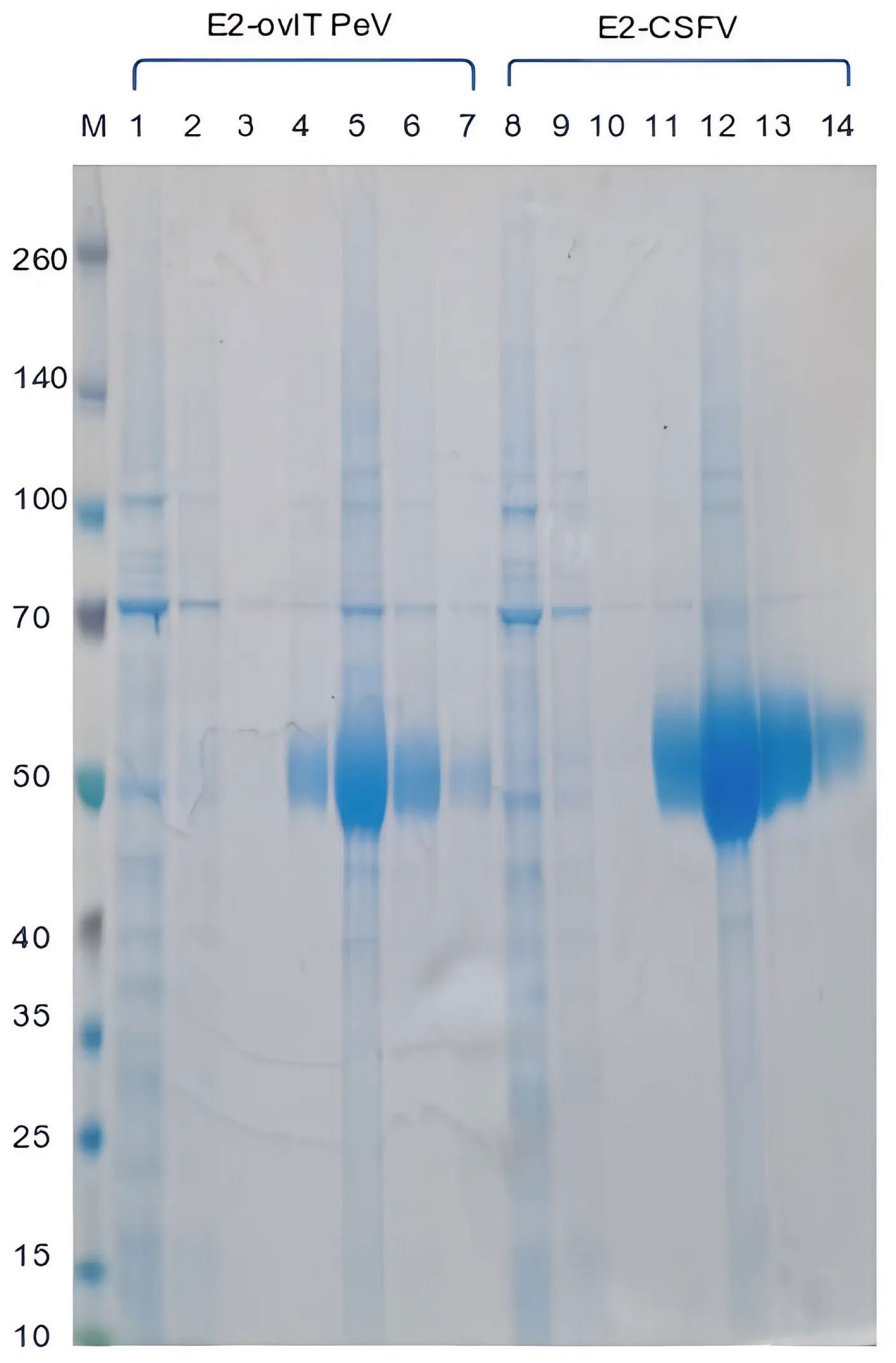
SDS-PAGE of the recombinant proteins purified after 3 days post-transfection. Lanes 1–7: FT, W1, W2, E1, E2, E3 and E4 of E2-ovIT PeV; Lanes 8–14: FT, W1, W2, E1, E2, E3 and E4 of E2-CSFV. M: molecular weight marker (kDa); FT: flow through after loading the antigen solution on the column; W1, W2: first and second wash steps to remove non-specifically bound proteins; E1–E4: first to fourth elution fractions containing the recombinant protein.

The optimized E2-ovIT PeV and E2-CSFV nucleotide sequences were cloned and transfected into two distinct suspensions of Expi293F cell cultures. At three and 6 days after plasmid transfection, the culture supernatants of E2-ovIT PeV and E2-CSFV were collected and the proteins were purified with IMAC using a nickel-charged resin with a high affinity for his-tagged proteins. As shown in Figure 2, proteins of approximately 50–55 kDa weight were eluted, correlating with 45 kDa, considering the amino acid composition and the presumptive post-translational modifications. Using 9 × 10^7^ Expi293F cells, 2.36 mg of the E2-CSFV and 1.4 mg of E2-ovIT PeV were obtained after purification.

The purified proteins were analyzed by WB using an anti-his primary antibody (Figure 1A) and pathogen-specific positive and negative porcine sera (Figures 1B,C).

The anti-his antibody detected both recombinant proteins with a molecular weight of 50–55 kDa. The same results were obtained by using the homologous pathogen-specific positive sera, while the negative sera did not show any reactivity (Figure 1).

### Preliminary serological evaluation of the tested sera

All sera evaluated in the present study were tested in two ELISA assays: a Pan-Pestivirus competitive NS3 ELISA and a commercial E2-CSFV competitive ELISA (Supplementary Table 1). The obtained results confirmed that the 339 field sera were negative, while all sera derived from experimental infections of pigs with CSFV tested positive in both assays. Nine out of 11 sera from experimental infection with the ovIT PeV scored positive in both assays whereas one sample was negative (sample number: ovIT PeV-1-t1) and one was classified as doubtful (sample number: ovIT PeV-6-t1) in the commercial E2 CSFV ELISA.

### Evaluation of iELISA

**Figure 3:**
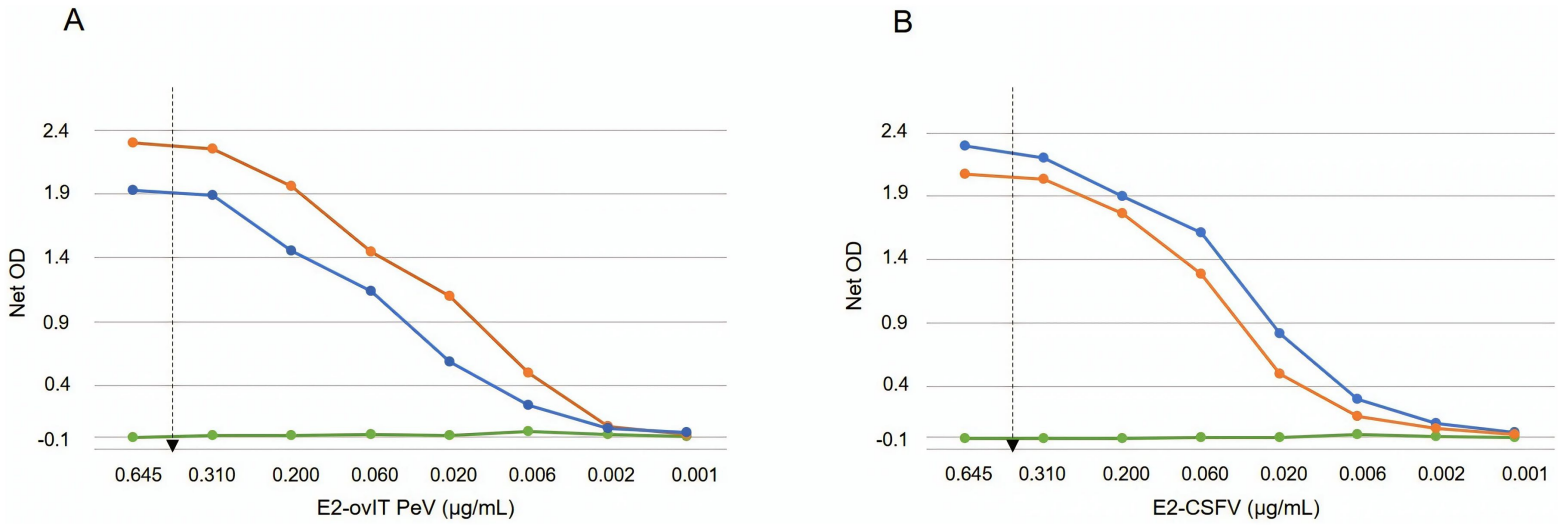
Titration of the antigens E2-ovIT PeV **(A)** and E2-CSFV **(B)** using pathogen-specific positive and negative sera. Orange curves represent the reaction of the antigens with the ovIT PeV antibody positive serum, while blue curves represent the reaction of the antigens with the CSFV antibody positive serum. The pestivirus antibody negative serum was applied for both antigens (green curves). The determined antigen concentration of the recombinant proteins (0.4 μg/mL) for coating is indicated by a dotted vertical lane.

For both proteins, an antigen concentration of 0.4 μg/mL was used for coating of the ELISA plates. This concentration gave a plateau signal after analysing a homologous and heterologous antibody positive serum in the ELISA, which confirms the saturation of the plate wells by the antigen coating (Figure 3). The optimal dilution of the conjugated anti-swine IgG Mab 4B6 corresponded to the highest value of signal-to-blank ratio calculated for the positive sera tested.

To determine the cut-off of the two iELISAs, all the 339 pestivirus antibody negative sera were evaluated with both the iELISA tests, while ovIT PeV and CSFV antibody positive sera were used to determine the cut-off of the, respectively, homologous test. A serum dilution of 1:50 was considered to calculate the cut-off.

**Figure 4:**
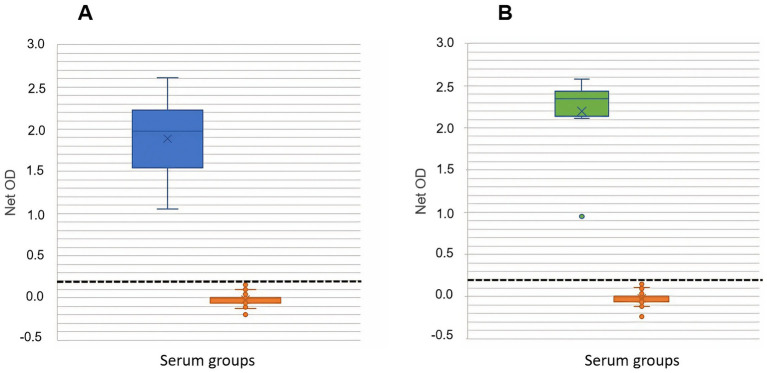
Net ODs distribution of the negative (orange), CSFV (blue) and ovIT PeV (green) antibody positive sera tested at 1/50 dilution in E2-CSFV iELISA **(A)** and in E2-ovIT PeV iELISA **(B)**. OD cut-off value of 0.2 is indicated by a dotted lane.

All 339 negative sera showed net OD values ranging between −0.240 and 0.198 in iELISA for ovIT PeV and between −0.195 and 0.182 in iELISA for CSFV (Figure 4) with 98% of the sera with OD values less than 0.1. Mean OD values were −0.021 ± 0.052 and −0.022 ± 0.044 for the E2-ovIT PeV and E2-CSFV iELISAs, respectively. Antibody positive ovIT PeV and CSFV sera tested against the homologous antigens showed net OD values distributed in the range from 0.984 to 2.575 and 1.056 to 2.609, with mean values of 2.195 ± 0.445 and 1.907 ± 0.458, respectively (Figure 4).

For both iELISAs the OD distribution showed a clear separation between positive and negative serawhen tested against their homologous antigens. Based on the observed distributions, a cut-off value of 0.2 OD was defined, corresponding approximately to the mean OD of negative sera plus three standard deviations. When tested with the E2-ovIT PeV iELISA the sera from CSFV infected animals showed positive results and the same occurred when the sera of pigs collected after infection with ovIT PeV were tested in E2-CSFV iELISA (data not shown).

Control sera included in each plate were used to monitor inter-plate consistency and assay performance. The CSFV-positive control serum showed a mean OD values of 2.179 ± 0.138 against E2-ovIT PeV (range: from 1.993 to 2.389) and 2.288 ± 0.106 against E2-CSFV (range: from 2.124 to 2.464). The negative control serum consistently showed low background reactivity, with mean OD values of −0.029 ± 0.059 against E2-ovIT PeV (range: from −0.180 to 0.114) and with mean OD values of −0.026 ± 0.057 (range: from −0.129 to 0.149) against E2-CSFV.

### Sera endpoint titration

**Figure 5:**
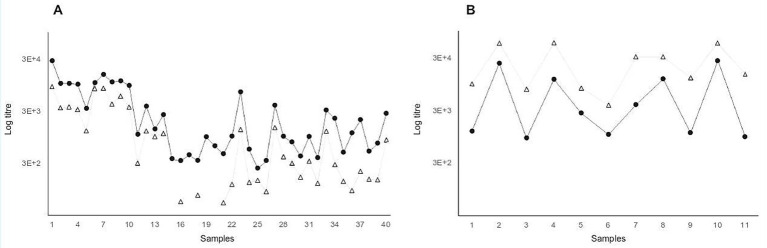
Graphical representation of the antibody titres expressed in logarithmic scale obtained by testing the sera collected from animals infected with CSFV **(A)** and ovIT PeV **(B)**. The titres obtained with E2-ovIT PeV iELISA are represented by an empty triangle, while the titers obtained with E2-CSFV iELISA are indicated by a full circle. No symbol is shown when the serum was negative. For sera from CSFV infected pigs the antibody titres against E2-CSFV were consistently higher than those against E2-ovIT PeV **(A)** (*p* < 0.001). The opposite was observed for the sera from pigs infected with ovIT PeV **(B)** (*p* < 0.001).

For each serum sample, antibody titres were determined using both developed iELISAs (18), and the results were compared (Figure 5). Consistently, all sera exhibited higher titres when tested against the homologous antigen than against the heterologous one with *p* < 0.001 according to Wilcoxon Signed Rank Test. Endpoint titres were determined in two independent experimental runs and showed good agreement between assays. Analysis of log-transformed titres revealed low inter-assay variability, with standard deviations of 0.16 and 0.20 for E2-ovIT PeV and E2-CSFV, respectively.

The analysis of the endpoint titer ratios (see Equation 2) showed that all samples with a ratio >1 (range: 1.29–12.93) were derived from pigs infected with CSFV, while those with a ratio <1 (range: 0.06–0.46) were classified as sera from pigs after ovIT PeV infection. Four out of 40 CSFV antibody positive sera showed no reactivity against the E2-ovIT PeV. Consequently, endpoint titres against E2-ovIT PeV could not be determined and the corresponding ratios could not be calculated.

Consistently with the titers obtained in iELISAs, all sera from animals obtained after infection with CSFV tested in VNT exhibited higher neutralizing antibody titers against the CSF0104 strain (Diepholz), with titers ranging from 1:80 to 1:7,680, while all sera from ovIT PeV infected animals showed the highest titers against the homologous ovIT PeV strain. However, for four antibody positive ovIT PeV samples (ovIT PeV-1-t2, ovIT PeV-3-t2, ovIT PeV-4-t2 and ovIT PeV-5-t2) the difference of neutralizing antibody titers was less than threefold when the VNT results were compared using the CSFV strain Diepholz and ovIT PeV. In particular, sample ovIT PeV-5-t2 exhibited identical titers against CSFV strain Diepholz and ovIT PeV.

## Discussion

The high genetic variability of pestiviruses has practical implications for their epidemiology, diagnosis, control and economic impact on livestock and their prevalence and presence is greater than actually recognised. In recent years new pestiviruses have been reported in domestic and non-domestic animals just as the atypical ovIT PeV (2, 11). Phylogenetic analysis showed that ovIT PeV and CSFV emerged from the same parental virus, the Tunisian sheep virus (TSV) in the beginning of the 19th century (8). Therefore, the genetic and antigenic similarities support a close relationship between these viruses and suggests their possible co-evolution as two branches stemming from a shared origin at the same time in two different hosts.

The close genetic relationship between ovIT PeV and CSFV presents significant challenges for disease control programs, particularly given that CSF is a notifiable disease to the World Organization for Animal Health (WOAH). In the event of widespread dissemination of ovIT PeV among ovine populations and potentially pigs, serious concerns arise. Experimental infection studies have demonstrated that ovIT PeV is capable of infecting pigs (12). Furthermore, the possibility of cross-reactivity in CSF antibody ELISA assays cannot be excluded, potentially complicating disease surveillance and diagnosis (12, 14).

While commercial pig farms implementing stringent biosecurity measures are expected to have a low risk of ovIT PeV transmission and associated complications, backyard farming systems pose a substantially higher risk. These systems often lack adequate biosecurity, and close contact between sheep and pigs facilitates viral spread. Additionally, contact between wild boars and sheep may serve as another pathway for transmission and dissemination of the virus. A surveillance program would benefit from the setup and validation of new methods able to detect and specifically discriminate antibodies induced by ovIT PeV from those induced by CSFV.

Serological analysis with regard to CSFV surveillance are routinely performed using commercially available CSF antibody ELISAs. With regard to serological cross-reactions after infection of pigs with ruminant pestiviruses, doubtful or positive results have to be confirmed by VNT using CSFV reference strains as well as reference strains of BVDV-1, BVDV-2, BDV, and additional ruminant pestiviruses circulating in the country. In such VNT assays, sera from pigs positive for antibodies against ruminant pestiviruses are expected to yield higher neutralization titres against the homologous virus strain compared to those obtained against CSFV. So far, natural infections of pigs with ruminant pestiviruses closely related to CSFV, such as Aydin pestivirus, Tunisian sheep pestivirus, and ovIT PeV, have not been reported. However, experimental infections showed that domestic pigs are susceptible to these viruses and confirmed a high level of serological cross-reactivity against CSFV. Cross-reactivity among pestiviruses may significantly compromise the specificity of serological assays, leading to difficulties in accurately distinguishing infections and representing a critical issue in the diagnosis and control of classical swine fever, given its severe sanitary and economic implications.

In this study we developed two novel indirect ELISA (iELISA) tests based on E2-ovIT PeV and E2-CSFV (Diepholz strain) recombinant proteins, respectively. The CSFV strain Diepholz was chosen since it showed the highest aminoacidic sequence identity with E2-ovIT PeV (10). Moreover, the viral E2 glycoprotein is highly immunogenic and represents the main target for the production of neutralizing antibodies after pestiviral infection (22). Both E2 proteins were efficiently expressed, secreted in the cell culture medium of the mammalian cells and purified by IMAC obtaining a good yield for each antigen. The SDS-PAGE and WB using anti-his Mab confirmed the expected molecular weight in the range of 50–55 KDa taking into account the presence of glycosylations and other post-traslational modifications. An ovIT PeV and a CSFV positive pooled sera used in two additional WBs, showed to react and correctly recognize the homologous recombinant protein.

First, all porcine sera included in the present study were analysed using a competitive Pan-Pestivirus ELISA test based on a non-structural protein NS3 and a commercial competitive ELISA specific for CSFV to confirm their serological status. This serum panel includes porcine samples from pestivirus negative animals and pigs that were experimentally infected with ovIT PeV and CSFV. Consistently, when tested in both competitive ELISAs, all pestivirus negative sera were confirmed as antibody negative and all CSFV samples resulted positive for antibodies against CSFV. The ovIT PeV sera were antibody positive in the NS3 ELISA, but nine out of eleven sera were false-positive in the CSF antibody ELISA.

When tested in our newly iELISAs, all pestivirus negative sera scored negative. However all samples from ovIT PeV infected pigs and the majority of samples from CSFV infected pigs were positive in both iELISA. In order to serologically distinguish the origin of the infection the endpoint titre for each serum in both E2-ovIT PeV and E2-CSFV iELISAs was calculated (18). The comparison of the titres showed that higher titres were obtained when the sera were tested in the homologous iELISA (*p* < 0.001).

To establish a parameter for distinguishing between CSFV and ovIT PeV infections, the ratio of E2-CSFV to E2-ovIT PeV end point titers was calculated for each serum. Only four CSFV antibody positive sera showed no reactivity in the E2-ovIT PeV iELISA, resulting in undefined ovIT PeV titers; therefore,the ratio could not be calculated for these samples, which were consequently classified as positive for CSFV antibodies.

All sera from CSFV infected pigs (40/40 CSFV antibody positive samples) exhibited a ratio higher than 1, ranging from 1.29 to 12.93, and were correctly classified as positive for CSFV antibodies. Conversely, all samples from ovIT PeV positive pigs (11/11 ovIT PeV antibody positive samples) showed a ratio below 1, ranging from 0.06 to 0.46, and were confirmed as positive for antibodies against ovIT PeV. The observed low inter-assay variability in both log-transformed titres and positive control sera indicates good reproducibility of the assay across independent experimental runs.

The results of the VNT carried out on selected sera are in accordance with the results obtained with the iELISAs. Analysis of the samples collected from pigs infected with ovIT PeV revealed that the titers of neutralizing antibodies against the CSFV strain Diepholz were substantially higher than those reported previously (12). Although VNT using specific virus variants should be able to show the virus against which the antibody response has been developed, in some cases is not conclusive (14).

The present study showed that for four ovIT PeV antibody positive samples (ovIT PeV-1-t2, ovIT PeV-3-t2, ovIT PeV-4-t2 and ovIT PeV-5-t2) a threefold titer difference between ovIT PeV and CSFV VNT was not detected. In this case, WOAH Terrestrial Manual recommends to re-sample the animals at a later time point, which might lead to an increase of antibody titers. In addition, further aspects must be included in the interpretation of the results (e.g., results of other CSF-specific tests, clinical signs of the animals, epidemiological situation).

Not all laboratories have the permission to work with infectious CSFV, which is a prerequisite conducting a VNT. However, these laboratories can perform ELISAs for the detection of antibodies. In particular, when CSF antibody ELISA detects positive sera or when infections with ovIT PeV are described in the country, the iELISAs of the present study offer an opportunity to test the samples with regard to ovIT PeV in comparison to CSFV. In general, according to the WOAH and EU Manuals for CSF Diagnostic, doubtful or positive CSF antibody ELISA results must be confirmed by VNT. In conclusion, the present study described in detail the close serological relationship of the ovIT PeV to CSFV and highlights the problem of cross-reactivity in CSF-specific serological tests, which emphasises the importance of confirmatory tests to rule out any CSFV infection. The application of the two newly developed iELISAs can provide initial indications of an infection with ovIT PeV, which must subsequently be confirmed by additional diagnostic tests. However, further analysis of porcine samples infected with a broader range of pestiviruses, including ruminant pestiviruses, is required to further assess assays specificity and validate the applicability of the proposed approach in different epidemiological contexts. In this perspective, the E2-based iELISA concept described in the present study could be expanded by including additional recombinant E2 antigens from other pestiviruses, potentially providing a flexible platform for the serological differentiation of infections caused by closely related pestiviruses.

## Data Availability

The original contributions presented in the study are included in the article/Supplementary material, further inquiries can be directed to the corresponding authors.

## Footnotes

1
https://arriveguidelines.org
