# Supplementary material for: Evaluation of an E2-based indirect ELISA for the serological differentiation of ovine Italy pestivirus from classical swine fever virus in pigs
Source: Front Vet Sci. 2026 Jun 16;13:1847235. doi: 10.3389/fvets.2026.1847235 (PMC13317440; doi:10.3389/fvets.2026.1847235)
Supplement: Supplementary file 1 [file Table_1.docx]

Supplementary Table 1. List of selected porcine sera from animals infected with ovIT PeV and various CSFV strains belonging to the indicated genotypes The table shows the sera inhibition percentages (IP) detected in the homemade Pan-Pestivirus NS3 competitive ELISA and the commercial IDEXX CSFV competitive ELISA (based on E2), the titres detected in the newly established iELISAs by using E2-ovIT PeV and E2-CSFV, and the neutralising antibody titers against CSFV, BVDV-1, BVDV-2, and ovIT PeV. t1: serum collected 28 dpi; t2: serum collected 35 dpi; *non competitive serum; **doubtful result; n.d. not determined; ***Sample ID of the EURL Classical Swine Fever Virus Database (24)

|  |  |  |  |  |  |  |  |  |  | | | |
| --- | --- | --- | --- | --- | --- | --- | --- | --- | --- | --- | --- | --- |
|  |  |  |  |  |  |  |  |  | **VNT assay [ND50]** | | | |
| **Sample number** | **Virus strain** | **ID***** | **Genotype** | **NS3 ELISA (IP)** | **IDEXX ELISA (IP)** | **E2-ovIT PeV titre** | **E2-CSFV titre** | **Ratio (E2-CSFV titre / E2-ovIT PeV titre)** | **CSFV (CSF0104 = Diepholz)** | **BVDV-1 (NADL)** | **BVDV-2 (CS8644)** | **ovIT PeV** |
| CSF-1 | Alfort 187 | CSF0902 | 1.1 | 97 | 73 | 9,148 | 28,660 | 3.13 | n.d. | n.d. | n.d. | n.d. |
| CSF-2 | Alfort 187 | CSF0902 | 1.1 | 97 | 74 | 3,597 | 10,455 | 2.91 | n.d. | n.d. | n.d. | n.d. |
| CSF-3 | Alfort 187 | CSF0902 | 1.1 | 97 | 79 | 3,725 | 10,455 | 2.81 | n.d. | n.d. | n.d. | n.d. |
| CSF-4 | Alfort 187 | CSF0902 | 1.1 | 96 | 85 | 3,337 | 10,101 | 3.03 | n.d. | n.d. | n.d. | n.d. |
| CSF-5 | Alfort 187 | CSF0902 | 1.1 | 96 | 87 | 1,300 | 3,466 | 2.67 | n.d. | n.d. | n.d. | n.d. |
| CSF-6 | Alfort 187 | CSF0902 | 1.1 | 97 | 74 | 8,393 | 10,807 | 1.29 | n.d. | n.d. | n.d. | n.d. |
| CSF-7 | Alfort 187 | CSF0902 | 1.1 | 96 | 75 | 8,525 | 15,601 | 1.83 | n.d. | n.d. | n.d. | n.d. |
| CSF-8 | Alfort 187 | CSF0902 | 1.1 | 97 | 81 | 4,220 | 11,190 | 2.65 | n.d. | n.d. | n.d. | n.d. |
| CSF-9 | Alfort 187 | CSF0902 | 1.1 | 96 | 87 | 6,008 | 11,729 | 1.95 | n.d. | n.d. | n.d. | n.d. |
| CSF-10 | Alfort 187 | CSF0902 | 1.1 | 96 | 89 | 3,709 | 9,572 | 2.58 | n.d. | n.d. | n.d. | n.d. |
| CSF-11 | Alfort 187 | CSF0902 | 1.1 | 75 | 84 | 313 | 1,094 | 3.50 | n.d. | n.d. | n.d. | n.d. |
| CSF-12 | Alfort 187 | CSF0902 | 1.1 | 96 | 85 | 1,294 | 3,816 | 2.95 | n.d. | n.d. | n.d. | n.d. |
| CSF-13 | Alfort 187 | CSF0902 | 1.1 | 95 | 86 | 1,010 | 1,384 | 1.37 | n.d. | n.d. | n.d. | n.d. |
| CSF-14 | Alfort 187 | CSF0902 | 1.1 | 95 | 82 | 1,158 | 2,615 | 2.26 | n.d. | n.d. | n.d. | n.d. |
| CSF-15 | Alfort 187 | CSF0902 | 1.1 | 77 | 66 | 0 | 372 | - | n.d. | n.d. | n.d. | n.d. |
| CSF-16 | Alfort 187 | CSF0902 | 1.1 | 92 | 74 | 55 | 340 | 6.18 | n.d. | n.d. | n.d. | n.d. |
| CSF-17 | Alfort 187 | CSF0902 | 1.1 | 88 | 64 | 0 | 442 | - | n.d. | n.d. | n.d. | n.d. |
| CSF-18 | Alfort 187 | CSF0902 | 1.1 | 91 | 80 | 73 | 344 | 4.71 | n.d. | n.d. | n.d. | n.d. |
| CSF-19 | Alfort 187 | CSF0902 | 1.1 | 87 | 75 | 0 | 985 | - | n.d. | n.d. | n.d. | n.d. |
| CSF-20 | Alfort 187 | CSF0902 | 1.1 | 88 | 58 | 0 | 656 | - | n.d. | n.d. | n.d. | n.d. |
| CSF-21 | Alfort 187 | CSF0902 | 1.1 | 70 | 65 | 52 | 462 | 8.88 | n.d. | n.d. | n.d. | n.d. |
| CSF-22 | Alfort 187 | CSF0902 | 1.1 | 95 | 60 | 119 | 1,012 | 8.50 | n.d. | n.d. | n.d. | n.d. |
| CSF-23 | Alfort 187 | CSF0902 | 1.1 | 96 | 83 | 1,371 | 7,196 | 5.25 | n.d. | n.d. | n.d. | n.d. |
| CSF-24 | Nep28/Brain/Makwanpur | CSF1059 | 2.2 | 94 | 54 | 129 | 563 | 4.36 | n.d. | n.d. | n.d. | n.d. |
| CSF-25 | CSF0940 (C-strain) | CSF0940 | 1.1 | 93 | 66 | 141 | 244 | 1.73 | n.d. | n.d. | n.d. | n.d. |
| CSF-26 | 14598PGV5.B Spleen | CSF1055 | 2.1 | 77 | 60 | 86 | 342 | 3.98 | n.d. | n.d. | n.d. | n.d. |
| CSF-27 | Diepholz | CSF0104 | 2.3 | 96 | 84 | 1,506 | 3,972 | 2.64 | n.d. | n.d. | n.d. | n.d. |
| CSF-28 | Parma98 | CSF0573 | 2.1 | 96 | 74 | 412 | 1,001 | 2.43 | n.d. | n.d. | n.d. | n.d. |
| CSF-29 | A-2 | CSF1048 | 2.1 | 96 | 75 | 313 | 781 | 2.50 | n.d. | n.d. | n.d. | n.d. |
| CSF-30 | 3795/96 | CSF0375 | 1.2 | 93 | 93 | 163 | 417 | 2.56 | 120 | 5 | <10 | 20 |
| CSF-31 | Congenital Tremor | CSF0410 | 3.1 | 96 | 90 | 340 | 995 | 2.93 | 240 | <5 | <10 | 30 |
| CSF-32 | PR, VP32/10 | CSF1058 | 1.4 | 88 | 93 | 125 | 388 | 3.10 | 80 | <5 | <10 | <20 |
| CSF-33 | Nep28/Brain/Makwanpur | CSF1059 | 2.2 | 94 | 95 | 1,277 | 3,202 | 2.51 | 1,280 | 40 | <10 | 240 |
| CSF-34 | Kanagawa (Tap3) | CSF0309 | 3.4 | 96 | 95 | 294 | 2,230 | 7.59 | 640 | 60 | <10 | <20 |
| CSF-35 | Diepholz | CSF0104 | 2.3 | 95 | 92 | 136 | 493 | 3.63 | 640 | <5 | <10 | 80 |
| CSF-36 | Alfort 187 | CSF0902 | 1.1 | 96 | 93 | 90 | 1,164 | 12.93 | 80 | <5 | <10 | <20 |
| CSF-37 | Guatemala HC/ #4409 | CSF0650 | 1.3 | 96 | 97 | 211 | 2,093 | 9.92 | 1,280 | 40 | 10 | 120 |
| CSF-38 | A-2 | CSF1048 | 2.1 | 96 | 97 | 149 | 517 | 3.47 | 2,560 | 15 | <10 | 60 |
| CSF-39 | 7045/2 | CSF1042 | 2.3 | 95 | 97 | 145 | 739 | 5.10 | 960 | <5 | <10 | 80 |
| CSF-40 | Diepholz | CSF0104 | 2.3 | 96 | 98 | 866 | 2,776 | 3.21 | 7,680 | 80 | 80 | 960 |
| ovIT PeV-1-t1 | IT/ov/1756/2017 | - | - | 93 | 27* | 9,823 | 1,232 | 0.13 | 80 | 20 | <10 | 240 |
| ovIT PeV-1-t2 | IT/ov/1756/2017 | - | - | 97 | 81 | 58,153 | 24,138 | 0.42 | 2,560 | 40 | 10 | 3,840 |
| ovIT PeV-2-t1 | IT/ov/1756/2017 | - | - | 97 | 50 | 7,792 | 914 | 0.12 | 80 | 10 | <10 | 960 |
| ovIT PeV-2-t2 | IT/ov/1756/2017 | - | - | 97 | 73 | 59,165 | 11,996 | 0.20 | 1,280 | 960 | 160 | 15,360 |
| ovIT PeV-3-t1 | IT/ov/1756/2017 | - | - | 97 | 75 | 8,174 | 2,727 | 0.27 | 80 | 10 | <10 | 320 |
| ovIT PeV-3-t2 | IT/ov/1756/2017 | - | - | 97 | 58 | 3,885 | 1,068 | 0.33 | 2,560 | 240 | 10 | 3,840 |
| ovIT PeV-4-t1 | IT/ov/1756/2017 | - | - | 97 | 50 | 31,968 | 3,941 | 0.12 | 120 | 15 | <10 | 640 |
| ovIT PeV-4-t2 | IT/ov/1756/2017 | - | - | 97 | 69 | 31,959 | 12,150 | 0.38 | 7,680 | 320 | 15 | 10,240 |
| ovIT PeV-5-t1 | IT/ov/1756/2017 | - | - | 96 | 69 | 12,813 | 1,151 | 0.09 | 120 | 10 | <10 | 3,840 |
| ovIT PeV-5-t2 | IT/ov/1756/2017 | - | - | 97 | 78 | 58,512 | 27,073 | 0.46 | 7,680 | 240 | 30 | 7,680 |
| ovIT PeV-6-t1 | IT/ov/1756/2017 | - | - | 97 | 35** | 14,962 | 962 | 0.06 | 60 | <5 | <10 | 480 |
|  |  |  |  |  |  |  |  |  |  |  |  |  |
