# Supplementary figures and images for: Evaluation of an E2-based indirect ELISA for the serological differentiation of ovine Italy pestivirus from classical swine fever virus in pigs
Source: Front Vet Sci. 2026 Jun 16;13:1847235. doi: 10.3389/fvets.2026.1847235 (PMC13317440; doi:10.3389/fvets.2026.1847235)

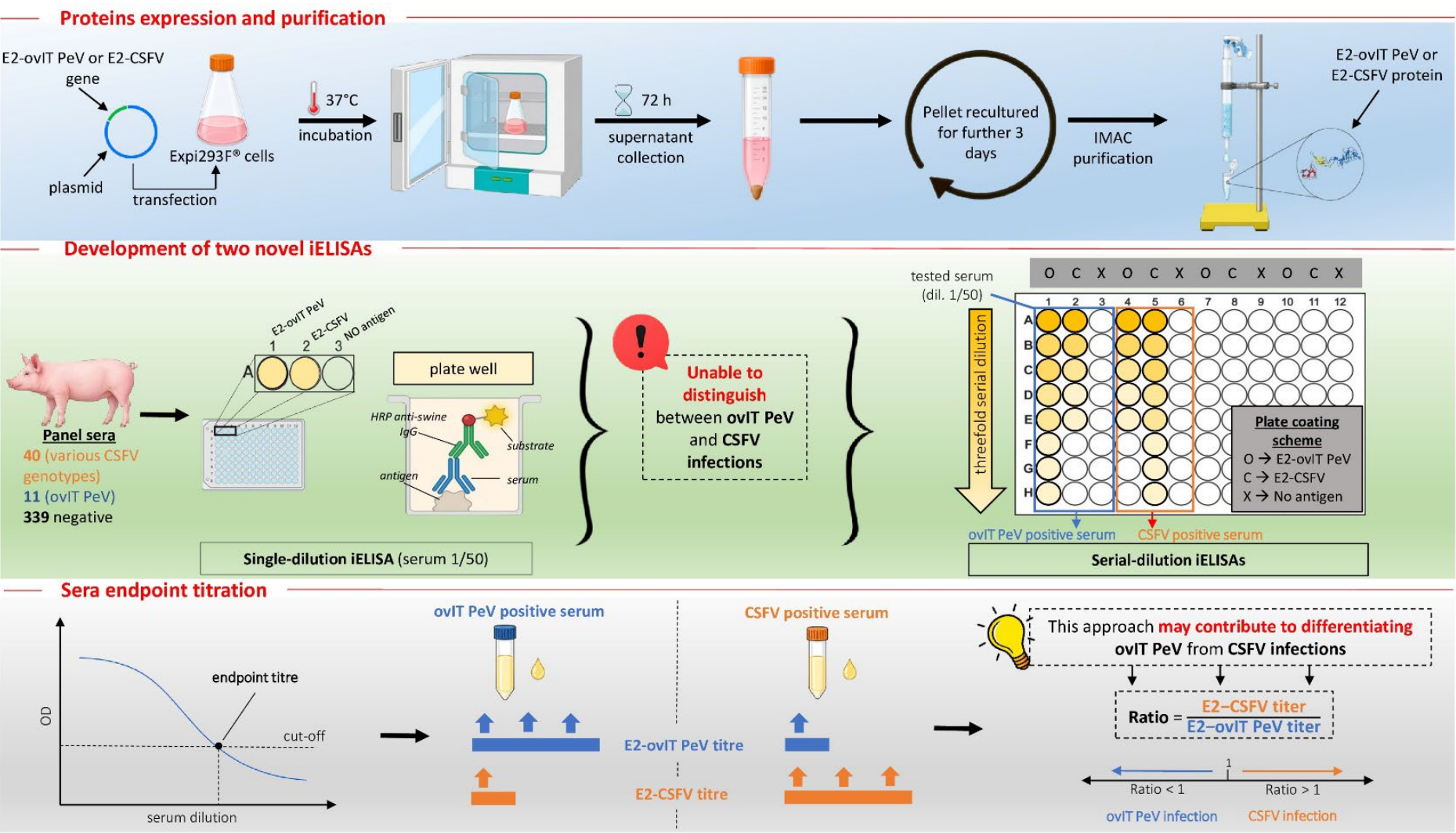

1 **Supplementary Figure 1.** Schematic overview of the overall experimental workflow

Supplement: Supplementary file 2 [file Image_1.pdf]
